# GABA_B_ Receptors Expressed in Human Aortic Endothelial Cells Mediate Intracellular Calcium Concentration Regulation and Endothelial Nitric Oxide Synthase Translocation

**DOI:** 10.1155/2014/871735

**Published:** 2014-07-09

**Authors:** Xu-Ping Wang, Zhen-Ying Cheng, Katrina L. Schmid

**Affiliations:** ^1^The Key Laboratory of Cardiovascular Remodeling and Function Research, Chinese Ministry of Education and Chinese Ministry of Health, Qilu Hospital, Shandong University, Jinan, Shandong 250012, China; ^2^Department of Ophthalmology, Qilu Hospital, Shandong University, Jinan, Shandong 250012, China; ^3^School of Optometry and Vision Science, Faculty of Health, and Institute of Health and Biomedical Innovation, Queensland University of Technology, Brisbane, QLD 4059, Australia

## Abstract

GABA_B_ receptors regulate the intracellular Ca^2+^ concentration ([Ca^2+^]*i*) in a number of cells (e.g., retina, airway epithelium and smooth muscle), but whether they are expressed in vascular endothelial cells and similarly regulate the [Ca^2+^]*i* is not known. The purpose of this study was to investigate the expression of GABA_B_ receptors, a subclass of receptors to the inhibitory neurotransmitter *γ*-aminobutyric acid (GABA), in cultured human aortic endothelial cells (HAECs), and to explore if altering receptor activation modified [Ca^2+^]*i* and endothelial nitric oxide synthase (eNOS) translocation. Real-time PCR, western blots and immunofluorescence were used to determine the expression of GABA_B1_ and GABA_B2_ in cultured HAECs. The effects of GABA_B_ receptors on [Ca^2+^]*i* in cultured HAECs were demonstrated using fluo-3. The influence of GABA_B_ receptors on eNOS translocation was assessed by immunocytochemistry. Both GABA_B1_ and GABA_B2_ mRNA and protein were expressed in cultured HAECs, and the GABA_B1_ and GABA_B2_ proteins were colocated in the cell membrane and cytoplasm. One hundred *μ*M baclofen caused a transient increase of [Ca^2+^]*i* and eNOS translocation in cultured HAECs, and the effects were attenuated by pretreatment with the selective GABA_B_ receptor antagonists CGP46381 and CGP55845. GABA_B_ receptors are expressed in HAECs and regulate the [Ca^2+^]*i* and eNOS translocation. Cultures of HAECs may be a useful *in vitro* model for the study of GABA_B_ receptors and vascular biology.

## 1. Introduction

GABA_B_ receptors, a distinct subclass of receptors to the inhibitory neurotransmitter *γ*-aminobutyric acid (GABA), comprised of two principal heterodimeric subunits GABA_B1_ and GABA_B2_ [1–3], are members of the metabotropic receptor family that via Gi/o proteins interact with neuronal inwardly rectifying potassium and voltage-gated calcium channels and when activated mediate slow synaptic inhibition [4]. GABA_B_ receptors are mainly located within the central nervous system and retina [5, 6] and modulate intracellular calcium concentration ([Ca^2+^]*i*) in selected neural cells (e.g., chromaffin cells [7], dopaminergic neurons [8], and cortical neurons [9]). GABA_B_ receptors have also been detected in peripheral tissues, including human and guinea pig airway epithelium [10], human fallopian tube [11], and human airway smooth muscle [12], and shown to participate in the [Ca^2+^]*i* modulation [13].

Vascular endothelial cells are principal cells of blood vessels and play crucial roles within the vasculature. Intracellular Ca^2+^ contributes to vascular endothelium physiology, functions, and disorders such as proliferation [14], apoptosis [15], permeability [16], endothelial nitric oxide synthase (eNOS) activation [17], injury [18], and healing [19]. Other neural-transmitter receptor types that are generally thought of as having primarily central neural system locations and functions have been shown to be present within the peripheral vascular endothelium cells and modify the intracellular Ca^2+^. For example, the muscarinic receptor subtypes 1 and 3 were detected in human vascular endothelial cells [20]. Acetylcholine increases the [Ca^2+^]*i* in primary cultured rabbit aortic endothelial cells, and this can be blocked by the selective muscarinic receptor antagonist atropine [21]. *β*-Adrenoceptors are present in the endothelium of the rabbit coronary artery [22]. Epinephrine induces endothelial Ca^2+^ influx and thus increases the [Ca^2+^]*i* in primary cultured bovine aortic endothelial cells and this can be inhibited by a *β*2-adrenoceptor antagonist ICI-118551 [23]. 5-Hydroxytryptamine (5-HT)1D, 5-HT2B, and 5-HT4 receptors are expressed in human umbilical vein endothelial cells [24, 25]. 5-HT stimulates Ca^2+^ uptake and this can be inhibited by 5-HT receptor antagonists [26].

As the major source of nitric oxide (NO) in vascular endothelial cells [27], endothelial nitric oxide synthase (eNOS) plays a crucial role within the cardiovascular system. The subcellular location of eNOS contributes to the enzyme functions [27]. In resting endothelial cells, eNOS is mainly located at the cell membrane and cytoplasm, and when stimulated by agonists, it translocates to structures within the cell cytosol close to the nucleus [27, 28]. eNOS translocation can be induced by a variety of agents, some of which stimulate the [Ca^2+^]*i* increase in endothelial cells. For example, acetylcholine [21, 29], endothelin-1 [30, 31], platelet-activating factor [29, 32], bradykinin [28], estrogen [33], and epicatechin [34] increase the [Ca^2+^]*i* in endothelial cells and induce eNOS translocation. Thus, GABA_B_ receptors regulate the [Ca^2+^]*i* in some neural and nonneural cells [7–9, 13]; whether they are expressed in vascular endothelial cells and regulate the [Ca^2+^]*i* and eNOS translocation is not clear.

Based on indirect evidence, we hypothesized that GABA_B_ receptors would be expressed in human aortic endothelial cells (HAECs). The purpose of this study was to investigate whether GABA_B_ receptors are expressed in cultured HAECs and regulate the [Ca^2+^]*i* and eNOS translocation. If these receptors are present, HAECs could be a useful model for studying a direct role of GABA in vascular regulation.

## 2. Methods

### 2.1. Cell Culture

Primary HAECs obtained from the American Type Cell Collection (VA, USA) were cultured in endothelial cell medium (ECM) containing 5% FBS and 1% endothelial cell growth supplement (ScienCell, USA) at 37°C with humidified air and 5% CO_2_. HAECs of no more than passage 4 were used. The study was carried out in accordance with “The Code of Ethics of the World Medical Association (Declaration of Helsinki)” for experiments involving humans.

### 2.2. Real-Time PCR

RNA isolation and reverse transcription were performed as previously described [35]. RNA concentration and purity were determined at an optical density ratio of 260 : 280 using a spectrophotometer. Primers for human GABA_B1_ and GABA_B2_ were designed to span a region that includes an intron in the genomic sequence for these genes and ordered from Shanghai Biosune Biotechnology Company (Shanghai, China). The primers for GABA_B1_ were forward 5′-GCCGCTGTGTCCGAATCTGCT-3′ and reverse 5′-CTGCGCGCCGTTCTGAGTGT-3′, and for GABA_B2_ they were forward 5′-TGGAGGCGTCTGTCCATCCGT-3′ and reverse 5′-GTCTTGCGTCAGCGTGCCCA-3′. SYBR Green real-time PCR and quantitative assays were performed by use of a Real-time PCR Detection System, LightCycler (Roche Applied Science, IN, USA). Denaturation was performed for 10 s at 95°C, annealing for 10 s at 60°C, and extension for 10 s at 72°C. cDNA from the human retinal tissue was used as the positive control and the samples without cDNA were used as the negative control. *β*-Actin was used as the housekeeping gene. Correct product size (228 bp for GABA_B1_ and 220 bp for GABA_B2_) was confirmed by DNA agarose gel, and sequence comparison with target genes was conducted (by Biosune Biotechnology Company, Shanghai). Samples were analyzed in triplicate.

### 2.3. Immunocytochemistry

Immunocytochemistry was performed as previously described [36]. Briefly, cells were fixed with 4% paraformaldehyde, blocked with 10% normal donkey serum, and incubated with mouse anti-human GABA_B1_ antibody (1 : 500; Abcam, USA), goat anti-human GABA_B2_ antibody (1 : 100; Santa Cruz, CA, USA), or rabbit anti-human eNOS antibody (1 : 100; Sigma, USA) overnight at 4°C. Subsequently, the cells were incubated with donkey anti-mouse secondary antibody (Alexa 568 conjugated; 1 : 1000; Invitrogen, CA, USA), donkey anti-goat secondary antibody (Alexa 488 conjugated; 1 : 1000; Invitrogen, CA, USA), and donkey anti-rabbit secondary antibody (Alexa 488 conjugated; 1 : 1000; Invitrogen, CA, USA) for 1 h at 37°C. A drop of Prolong Gold anti-fade reagent with DAPI (Invitrogen, CA, USA) was added before cell images were acquired by use of a LSM 710 laser confocal microscope (EC Plan-Neofluar 40×/1.30 Oil objective, N.A. 0.55) equipped with ZEN 2009 Light Edition software (Zeiss, Germany).

### 2.4. Western Blots

Western blots were performed as previously described [35, 36]. HAECs and human retinal tissue (positive control) protein samples were separated by 7.5% SDS-PAGE and transferred to polyvinylidene difluoride (PVDF) membranes. After 1 h defatted milk block, the membrane was incubated with the anti-GABA_B1_ antibody (Abcam, 1 : 1000) and anti-GABA_B2_ antibody (Santa Cruz; 1 : 200) and then with horseradish peroxidase- (HRP-) conjugated secondary antibody (1 : 5000). The bands were visualized by use of Immobilon Western Chemiluminescent HRP Substrate (Millipore, MA, USA).

### 2.5. Measuring [Ca^2+^]*i*

[Ca^2+^]*i* was measured as previously described [37] with minor modification. HAECs were cultured on a glass-covered disc with a concentration of 5 × 10^5^ cells/mL. Two days later, the cells were incubated with 5 *μ*M fluo-3 AM for 20 min in the dark in normal physiological saline solution (N-PSS) that contained (in mM) 140 NaCl, 1 KCl, 1 CaCl_2_, 1 MgCl_2_, 10 glucose, and 5 HEPES (pH 7.4) at 37°C. After being rinsed twice with N-PSS, cells were kept in N-PSS for another 10 min, and then the circular discs with HAECs attached were placed on the stage of a confocal microscope. While the images were being acquired, 100 *μ*M agonist baclofen was added to the assay disc at the set time points. To determine the impact of the antagonist, cells were preincubated for 10 min with 1 mM of the GABA_B_ receptor antagonists CGP46381 (Santa Cruz, CA, USA) and CGP55845 (Tocris Bioscience, USA) before 100 *μ*M baclofen (Sigma, MO, USA) was added. Sequences of images were acquired using the laser confocal microscope (LSM 710, Zeiss, Germany) equipped with a 488 nm laser at 5 s intervals. The fluorescence intensity over the HAECs cell body was measured before and after agent application. The fluorescence intensity before addition of agents was considered the baseline fluorescence intensity. The changes of fluorescence intensity after agent application were calculated and analyzed by using ZEN 2009 Light Editin software (Zeiss, Germany). PBS instead of GABA_B_ agents were used as the control.

### 2.6. eNOS Translocation

eNOS translocation was investigated using immunofluorescence as previously described [33] with minor modification. To test the effects of the GABA_B_ receptor agonist on eNOS translocation, cells were treated with 100 *μ*M baclofen for 30 min. To determine the impact of the antagonist, cells were preincubated for 10 min with 1 mM of the GABA_B_ receptor antagonists CGP46381 and CGP55845 before 100 *μ*M baclofen was added.

### 2.7. Statistical Analysis

All data were analyzed using SPSS v16.0 (SPSS Inc., Chicago, IL, USA). Data were acquired from at least 3 independent repeats of the experiments and were expressed as mean ± SD.

## 3. Results

**Figure 1:**
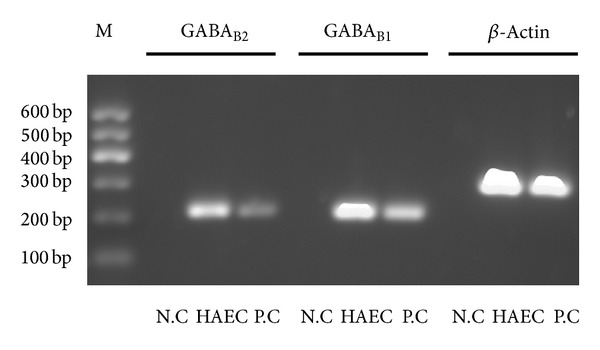
Expression of GABA_B1_ and GABA_B2_ mRNA in HAECs. Sample ethidium bromide gel of real-time PCR products of GABA_B1_ and GABA_B2_ receptors in HAECs and retina. M: DNA marker; GABA_B1_: GABA_B_ receptor subunit 1 (220 bp); GABA_B2_: GABA_B_ receptor subunit 2 (228 bp). N.C: negative control; HAECs: human aortic endothelial cells; P.C: human retina (positive control).

Real-time PCR demonstrated the presence of GABA_B1_, GABA_B2_, and *β*-actin mRNA in cultured HAECs and in human retina, but not in the negative control. Results from ethidium bromide-stained agarose gels electrophoresis of the real-time PCR products demonstrated that the specific bands appeared at the position of 228 bp (GABA_B1_), 220 bp (GABA_B2_), and 302 bp (*β*-actin), respectively (Figure 1). Nucleotide sequence analysis confirmed that the sequence of the PCR products is corresponding to the targeted sequence of GABA_B1_ and GABA_B2_ mRNA with the primers.

### 3.1. GABA_B1_ and GABA_B2_ Protein Were Detected in Cultured HAECs

**Figure 2:**
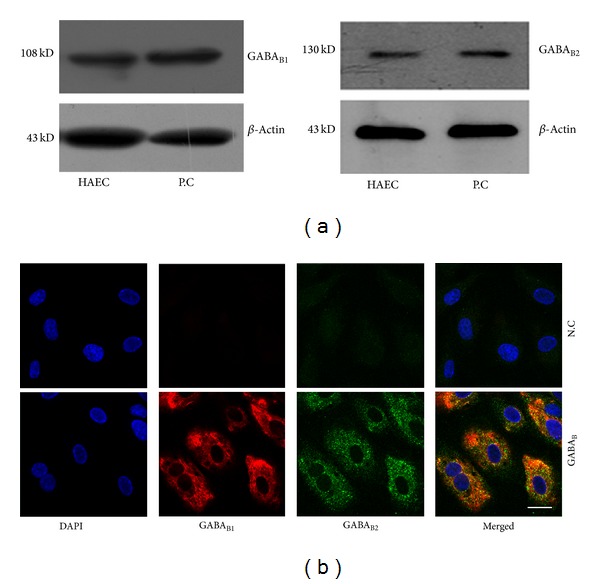
Expression of GABA_B1_ and GABA_B2_ protein detected by western blots (a) and immunofluorescence (b). (a) Specific bands developed at the approximate location of 108 kD (GABA_B1_), 130 kD (GABA_B2_), and 43 kD (*β*-Actin) in lysates of HAECs and retina (P.C). (b) Representative dual immunofluorescence staining of GABA_B1_ and GABA_B2_ in HAECs. Nuclei were stained by DAPI (blue) (scale bar = 50 *μ*m). HAECs: human aortic endothelial cells; P.C: human retina (positive control).

Western blots analysis revealed that specific protein bands appeared at approximately 108 kDa (GABA_B1_), 130 kDa (GABA_B2_), and 43 kDa (*β*-actin) (Figure 2(a)). Immunoreactivities to antibodies for GABA_B1_ and GABA_B2_ were observed in cultured HAECs. Immunofluorescence was observed in the cell membrane and cytoplasm but not in the nucleus (Figure 2(b)). These data confirmed the expression of GABA_B1_ and GABA_B2_ receptor protein in HAECs.

### 3.2. GABA_B_ Receptors Regulate [Ca^2+^]*i* in Cultured HAECs

**Figure 3:**
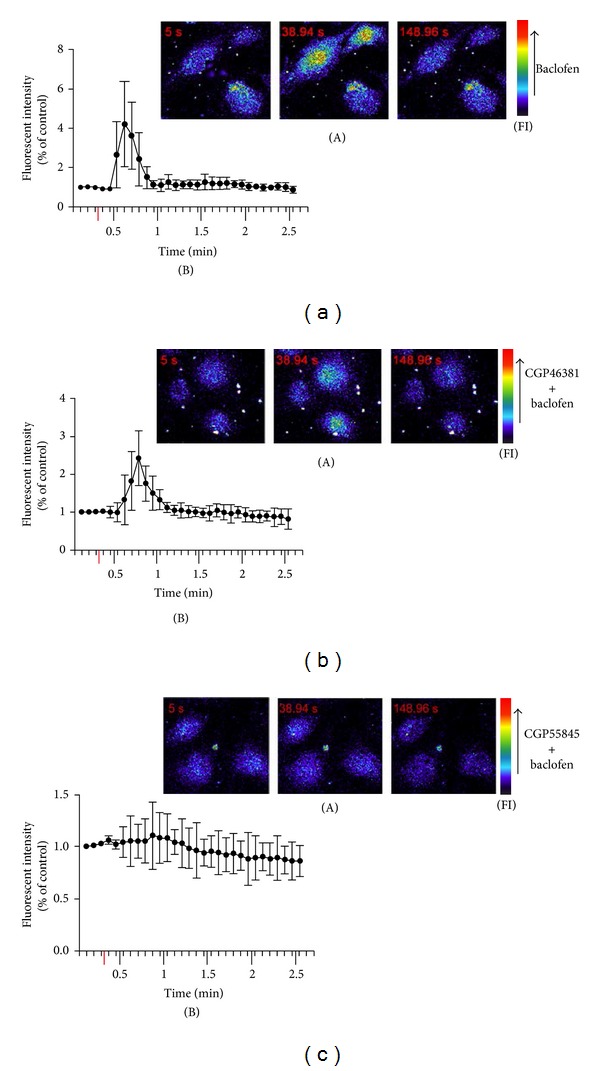
GABA_B_ receptors regulate [Ca^2+^]*i* in HAECs. (a) 100 *μ*M baclofen caused increase of [Ca^2+^]*i* in the cultured HAECs. (b) Pretreatment with 1 mM CGP46381 partly (~50%) abolished the increase of [Ca^2+^]*i* induced by 100 *μ*M baclofen. (c) Pretreatment with 1 mM CGP55845 completely inhibited the increase of [Ca^2+^]*i* induced by 100 *μ*M baclofen. (A) Representative images acquired at 5 s, 38.94 s, and 148.96 s, respectively. (B) Fluorescent intensity in the images acquired before and after baclofen application. The red bar represents the time when baclofen was added. FI represents the fluorescence intensity color scale with the direction of the arrow indicating higher intensity.

The GABA_B_ receptor agonist baclofen (100 *μ*M) induced a rapid and transient rise of [Ca^2+^]*i* in HAECs. [Ca^2+^]*i* reached its peak level in 20–40 s and then gradually declined (Figure 3(a)). The increase of [Ca^2+^]*i* induced by 100 *μ*M baclofen was partly (~50%) abolished by preincubation with 1 mM CGP46381 (Figure 3(b)) and was completely inhibited by preincubation with 1 mM CGP55845 (Figure 3(c)). PBS did not cause increase in [Ca^2+^]*i* of the cultured HAECs.

### 3.3. GABA_B_ Receptors Modulate eNOS Translocation in Cultured HAECs

**Figure 4:**
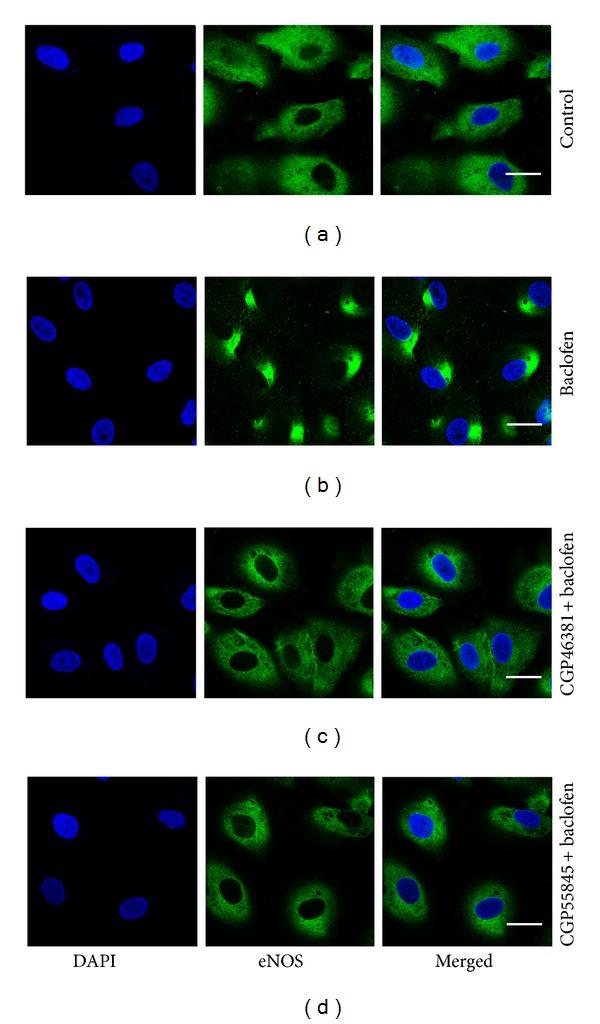
GABA_B_ receptors regulate eNOS translocation in HAECs. (a) In control HAECs, eNOS immunostaining was predominantly located at the cell membrane and cytoplasm. (b) 100 *μ*M baclofen incubated HAECs (incubation for 30 min) showed that eNOS immunostaining location was changed to intracellular sites close to the nucleus. (c) Preincubation of CGP46381 for 10 min inhibited 100 *μ*M baclofen induced translocation of eNOS in HAECs. (d) Preincubation of CGP55845 for 10 min inhibited 100 *μ*M baclofen induced translocation of eNOS in HAECs. Images are at ×400 magnification. Scale bar = 20 *μ*m.

In control HAECs, eNOS immunostaining was predominantly located at the cell membrane and cytoplasm (Figure 4(a)). One hundred *μ*M baclofen incubated HAECs (incubation for 30 min) showed that eNOS immunostaining was changed to intracellular sites close to the nucleus (Figure 4(b)). Preincubation of CGP46381 and CGP55845 for 10 min inhibited 100 *μ*M baclofen induced translocation of eNOS in HAECs (Figures 4(c) and 4(d)).

## 4. Discussion

In the study we found that GABA_B1_ and GABA_B2_ mRNA and protein were expressed in cultured HAECs; the two subunits were colocated in the cell membrane and cytoplasm, but neither was located in the nucleus. The GABA_B_ receptor agonist baclofen induced a transient increase of [Ca^2+^]*i* and eNOS translocation and the effects were attenuated by the GABA_B_ receptor antagonists CGP46381 and CGP55845. These findings suggest that GABA_B_ receptors are expressed in cultured HAECs and regulate the [Ca^2+^]*i* and eNOS translocation.

Muscarinic receptors [20, 21], *β*-adrenoceptors [22, 23], and 5-HT receptors [24–26] that are primarily located in central neural system are also present within the peripheral vascular endothelial cells and modulate the [Ca^2+^]*i*. Here we add GABA_B_ receptors to the list. Whether GABA_B_ receptor functions involving intracellular Ca^2+^ are similar to those of other neural-transmitters requires further investigation. Possibilities include proliferation [14], apoptosis [15], permeability [16], and eNOS activation [17].

The [Ca^2+^]*i* changes in vascular endothelial cells have been reported to be involved in eNOS activity, which mainly include eNOS translocation and phosphorylation [17, 27]. eNOS translocation from the plasma membrane to subcellular locations contributes to eNOS functions, such as permeability [27], and thecell membrane-bound and Golgi-bound eNOS are considered to have the ability to release more basal NO than cytosolic eNOS [27]. We found that GABA_B_ receptors modify the eNOS translocation by moving eNOS from the cell membrane and cytoplasm to the cytoplasm closer to the nucleus. It is thus possible that GABA_B_ receptors in HAECs regulate NO production and modify vascular permeability [27].

GABA_B_ receptors have been reported to be involved in regulating vasculature functions, but the mechanisms are complex. In addition to the central neural system mechanisms [38–40], GABA_B_ receptors directly regulate the vasculature functions via a peripheral mechanism. The GABA_B_ receptor agonist, SKF-97541, induces vasodepression in the feline pulmonary vascular bed and these responses are attenuated after the administration of a GABA_B_ receptor antagonist, saclofen [41]. The GABA_B_ receptor agonist baclofen causes vasodilation in 50% of vessels in the rat retina; the vasodilation can be blocked by the GABA_B_ receptor antagonist 2-hydroxysaclofen [42]. Here we verified that GABA_B_ receptors are expressed and located in cultured HAECs and regulate [Ca^2+^]*i* and eNOS translocation. These suggested that vascular endothelial cells would be the potential targets for GABA_B_ receptors directly modulating vascular functions.

In summary, GABA_B1_ and GABA_B2_ mRNA and protein were expressed in cultured HAECs and GABA_B_ receptors modified [Ca^2+^]*i* and eNOS translocation; this suggests a possible role of GABA_B_ receptors in the mediation of HAECs functions. Further investigation is required regarding which specific HAEC functions (e.g., proliferation, apoptosis, and permeability) GABA_B_ receptors regulate.

## Abbreviations

GABA: *γ*-Aminobutyric acid
GABA_B1_: *γ*-Aminobutyric acid B receptor subunit 1
GABA_B2_: *γ*-Aminobutyric acid B receptor subunit 2
HAECs: Human aortic endothelial cells
EC: Endothelial cells
eNOS: Endothelial nitric oxide synthase
NO: Nitric oxide
RT-PCR: Real-time polymerase chain reaction.
